# The ulnar collateral ligament loading paradox between in-vitro and in-vivo studies on baseball pitching (narrative review)

**DOI:** 10.1080/23335432.2021.1916405

**Published:** 2021-04-28

**Authors:** Bart Van Trigt, Liset (W) Vliegen, Ton (Ajr) Leenen, DirkJan (Hej) Veeger

**Affiliations:** a Department of Biomechanical Engineering, Delft University of Technology, CD Delft, The Netherlands; b Department of Human Movement Sciences, Faculty of Behavioural and Movement Sciences, Vrije Universiteit Amsterdam, Amsterdam Movement Sciences, BT Amsterdam, The Netherlands

**Keywords:** UCL, elbow injury, overhead sports, musculoskeletal modelling, electromyography; Tommy John Surgery;

## Abstract

Ulnar collateral ligament (UCL) weakening or tears occur in 16% of professional baseball pitchers. To prevent players from sustaining a UCL injury, it is important to understand the relationship between the UCL properties and elbow stabilizers with the load on the UCL during pitching. In-vitro studies showed that the ultimate external valgus torque of 34 Nm would rupture the UCL, which is in apparent conflict with the reported peak valgus torques in pitching (40–120 Nm). Assuming both observations are correct, the question rises why ‘only’ 16 out of 100 professional pitchers sustain a UCL rupture. Underestimation of the effect of other structures in in-vivo studies is most likely the explanation of this mismatch because the calculated in-vivo torque also includes possible contributions of functional and structural stabilizers. In-vitro studies show that the flexor-pronator mass has the potential to counteract valgus torque directly, whereas the elbow flexor-extensor muscles combined with the humeroradial joint might have an indirect effect on valgus torque by increasing the joint compression force. Accurate experimental electromyography data and a more detailed (musculoskeletal)mechanical model of the elbow are needed to investigate if and to what extent the structural and functional stabilizers can shield the UCL during pitching.

## Introduction

Baseball pitching is a highly dynamic movement that shows high injury rates. Conte et al. (2001) reported that 48% of the injured players in Major League Baseball (MLB) were pitchers. The shoulder and elbow were found to be the most frequent injury sites, responsible for 29% and 22% of the disabled days, respectively. A study by Lyman et al. (2001) on 298 youth pitchers reported that over two seasons, 26% of the pitchers experienced elbow pain. In 68% of those, elbow pain was experienced on the medial side. Most of the time, this pain is related to ulnar collateral ligament (UCL) injuries. Overall, the prevalence of UCL reconstruction is 16% in professional baseball pitchers (Conte et al. 2015).

The elbow is usually described as a hinge joint, allowing flexion-extension. This hinge-like behaviour is because rotations in other directions, such as varus-valgus, are resisted by structures around the joint, with the joint shape, joint ligaments and joint-crossing muscles as the most important factors (Buffi et al. 2015).

The late cocking phase and acceleration phase of the pitching movement have been reported to be critical in terms of elbow load (Fortenbaugh et al. 2009). The elbow load in these phases is also high in other overhead sport motions like the tennis serve (Elliott et al. 2003). In these phases, the elbow encounters an external valgus torque, which imparts a compressive force on the lateral side and a tensile force on the medial side of the elbow. The UCL plays an essential role in resisting this external valgus torque.

Knowledge of UCL loading may be used to prevent overuse UCL injuries. In-vitro studies have investigated the role of the UCL and its different parts in resisting external valgus torque. These static in-vitro studies provide more insight into the function, biomechanical properties and the ultimate torque of the UCL ligament, but do not provide information about the UCL loading during the baseball pitch or other overhead sports motions. It is highly complex, if not impossible, to measure the direct load of the UCL during pitching in a non-invasive way. To our knowledge, no experimental study has been published which directly measured the UCL load. The closest to this have been inverse dynamic studies that quantified the external valgus torque around the elbow as an indication for UCL loading. Most likely, other structures around the elbow are also likely to resist the external valgus torque (Buffi et al. 2015), although more insight about the contribution of these structures is needed to understand the UCL injury risk.

The goal of this review is to provide an overview of what risk factors are related to UCL injuries, and to better understand the relationship between the UCL properties and elbow stabilizers with the load on the UCL during pitching, by combining the literature of in-vitro and in-vivo studies.

## Risk factors of UCL injury in pitching

### UCL injury risk factors in pitching

It is widely accepted that elbow injury results from overuse. High torques and forces in the joint stress the ligaments, and repetitive valgus overload from throwing may cause a micro-rupture. When overuse is sustained, and the body is unable to compensate, this can lead to attenuation or even tear of the UCL (Fortenbaugh et al. 2009; Sakiko Oyama 2012; Safran 1995; Schwab et al. 1980; Weber et al. 2014). Many epidemiological studies have looked into factors that influence elbow injury risk in pitching (Table 1) (Lyman et al. 2001; Olsen et al. 2006; Bushnell et al. 2010; Fleisig et al. 2011; Keller et al. 2016).

**Table 1.** An overview of five epidemiological studies predicting elbow pain, injury or surgery. Descriptive information of the different studies is provided about subjects, age, pitchers level, highest fastball speed, study design, data collection and statistical tests. The included predictors are fatigue, pitch count, ball speed, pitch-type percentage, body weight and body height. The table shows whether a positive (+), negative (−) or no significant (0) relationship between the predictor and higher pain/injury risk was found, with its corresponding odds ratio (OR). ^a^ OR for >600 compared to <300 pitches. ^b^ OR increased with a higher weight class. ^c^ OR decreased with greater height class. ± = standard deviation

**Study design**	Lyman et al. (2001)	Fleisig et al. (2011)	Olsen et al. (2006)	Keller et al. (2016)	Bushnell et al. (2010)
**Subjects**	298 pitchers	481 pitchers	95 injured pitchers 45 control pitchers	83 injured pitchers 83 matched control	9 injured 14 control pitchers
**Age (range)**	10.8 ± 1.2 (8–12)	12.0 ± 1.7	18.5 ± 1.5	28 ± 4.2	28 (20–30)
**Pitchers level**			High school/college	Professional, MLB	Professional
**Fastball speed (injured vs. control)**			88.3 vs. 82.7 mph	91.3 vs. 91.5 mph	89.22 vs. 85.22 mph
**Data collection**	2 season follow-up interviews after game and season	10-year follow-up Annual interview	1-year time period retrospective survey	2 years before and after surgery online data	3 seasons cohort study online data and disabled list
**Predicting**	Elbow pain	Elbow injury	Elbow surgery	UCL reconstruction	Elbow injury
**Statistical outcome**	Odds ratio	Odds ratio	t-test	t-test	t-test
**Predictor**					
**Fatigue**	+ (OR 5.94)		+		
**Pitch count**	+ pitches/year (OR 3.44/0.47*^a^*)	+ innings/year (OR 3.5)	+ months/year + games/year + innings/game + pitches/game + pitches/year		
**Ball speed**			+	0	+
**Pitch type percentage**			0	+	
**Body weight**	+ (OR 1.31–5.39^b^)		+		0
**Body height**	− (OR 0.79–0.35^c^)		+		0

Pitching with self-reported fatigue showed increased odds of elbow pain (Lyman et al. 2001). Olsen et al. (2006) reported that pitchers who underwent elbow surgery were more likely to experience arm pain or fatigue while pitching.

The number of pitches thrown per inning, game and season is frequently associated with higher injury risk. Olsen et al. (2006) showed that injured pitchers, before sustaining an injury, threw more months per year (8 versus 5), games per year (29 versus 19), innings per game (6 versus 4), pitches per game (88 versus 66) and pitches per year (2500 versus 1300) compared to the uninjured matched control group. Fleisig et al. (2011) found that pitchers who threw more than 100 innings a year were 3.5 times more likely to sustain an injury. In youth pitchers, it has been shown that throwing more than 600 pitches per season during games increased the odds of developing elbow pain by 3.4 times compared to throwing fewer than 600 pitches (Lyman et al. 2001).

Not surprisingly, as ball speed is by definition related to external load on segments, three studies found that ball speed is related to injury risk. A case–control study by Olsen et al. (2006) found a difference between injured and non-injured pitchers (88 versus 83 mph), as did Bushnell et al. (2010) (89 versus 85 mph). Next to adult pitchers, also youth pitchers show an association between ball speed and elbow pain (Kurokawa et al. 2020). Ball speed did not decline following return to the sport: Keller et al. (2016) compared ball speed of MLB pitchers before and after UCL reconstruction surgery with data from a matched control group with no injury history. No significant difference in ball speed between the groups was found.

Pitch type percentage (fastball, curveball, slider, etc.) is another risk factor that has been investigated in relation to injuries. Keller et al. (2016) reported that throwing more than 48% fastballs increased the UCL injury risk among professional players. In contrast, this was not supported by the study by Olsen et al. (2006) in which both control and injured college pitchers threw 61% fastballs. The absence of a correlation between percentage of fastballs and injury risk in Olsen’s study might be explained by the fact that the players were younger and that at lower level overall more fastballs are thrown (Table 1).

Body weight has been reported to increase injury risk by both Olsen et al. (2006) and Lyman et al. (2001). However, these studies do not agree on the influence of pitcher height: Olsen et al. (2006) found that an increased body height corresponded with higher injury risk, while Lyman et al. (2001) found that decreased height was a risk factor for injury. Theoretically, greater body height and weight would both increase the inertia of the forearm, leading to higher torques around the elbow. However, the stabilizing structures, as muscles, around the elbow might also be stronger in heavier or taller players. Therefore, body fat percentage might be an interesting risk factor to investigate in relation to elbow injuries.

Strength training is also an important aspect in pitching. Strength training might influence injury risk, since weight lifting during the season was found to increase the risk of elbow and shoulder pain in 8–12 years old pitchers (Lyman et al. 2001). However, this weight lifting was self-reported, which makes it unclear how the training was performed and whether it was conducted under supervision. In contrast, Sakata et al. 2017 found that medial elbow injuries in youth baseball pitchers were significantly lower in their intervention group. This intervention was more sports specific, with nine strength and stretch exercises, compared to the study of Lyman et al.(2001). It seems that strength training programs should focus on motor control to prevent elbow injuries. The effect of strength training in adults has not been investigated.

Lastly, it has been widely suggested that an ‘improper’ pitching technique can increase injury risk (Fortenbaugh et al. 2009; Oyama et al. 2014; Weber et al. 2014). Pitching technique can cause higher joint torques and forces. If knowledge is gained on what pitching technique leads to higher injury risk (what ‘improper’ pitching technique is), pitchers can adjust their technique in order to prevent injury.

Overall, fatigue and pitch count seem to be related to UCL injuries. The literature is not consistent about the relation between body weight and height, ball speed and pitch-type percentage in relation to UCL injuries. To understand the risk factors in relation to possible injury mechanisms, it is necessary to understand the behaviour of the UCL and other joint stabilizers during pitching.

## The difference in UCL and elbow load between in-vitro and in-vivo studies

### UCL load in in-vitro studies

**Figure 1.**
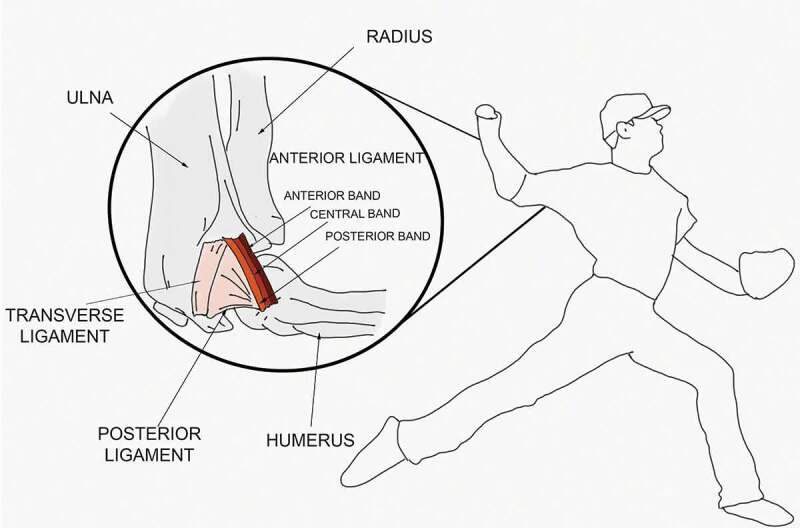
Anatomical sketch of the UCL during pitching. The UCL consists of the transverse ligament, posterior oblique ligament and anterior oblique ligament. The anterior oblique ligament contains three parts; the anterior, posterior and central band.*(This figure is inspired based on the figure made by Rik Molenaar)*

In-vitro studies showed that the UCL complex consists of three different ligaments: the anterior oblique ligament (AOL), the posterior oblique ligament (POL) and the transverse ligament (TL). Some studies refer to bundles instead of ligaments (Figure 1). According to Kaufmann et al. (2019), the primary stabilizer in resisting external valgus torque is the AOL, whereas the contribution of the POL is negligible, and the TL lacks the ability to resist valgus torque due to its origin and insertion on only the ulna (Kaufmann et al. 2019). The AOL can be further divided into the anterior band and the posterior band (Callaway et al. 1997; Jackson et al. 2016), and one study even refers to a third central band (Ciccotti et al. 2009) (Figure 1).

Several studies have investigated the mechanical properties of the AOL in-vitro, see Table 2 (Ahmad et al. 2003; Dillman 1991; Regan et al. 1991; Hechtman et al. 1998; McGraw et al. 2013). All studies showed an ultimate torque resistance strength of approximately 30 Nm. Ahmad et al. (2003) and McGraw et al. (2013) pre-loaded the cadavers to 1 Nm and then loaded them to failure. Both studies also calculated the stiffness: Ahmad et al. (2003) found a mean stiffness of 42.81 N/mm and McGraw et al. (2013) a mean stiffness of 21.0 N/mm. This substantial disparity might be explained by different elbow flexion angles (70 and 30 degrees), different loading rates (50% strain/s and 67% strain/s) and the properties of the cadavers (male versus both sexes, mean age 44 versus 52 years). Another study (Dillman 1991) estimated the UCL ultimate force by dividing the applied ultimate failure torque by an estimated moment arm. This approach has the drawback that the moment arm is actually unknown and might be influenced by testing conditions. Therefore, directly calculating the ultimate force of a ligament will provide more precise information about its mechanical properties. Regan et al. (1991) investigated the UCL strength by preparing bone-ligament-bone samples, which were preloaded and then loaded to failure with a loading rate of 100% of the initial length per second. They determined a failure load of 260.9 N and stiffness of 1528 N for the AOL. Comparable values were found by Jackson et al. (2016), who found a failure load of 293.1 N for the AOL and a mean yield point of 203.3 N.

**Table 2.** Mechanical properties of the UCL in different in-vitro studies. ± = standard deviation

	Number of specimens (m = male, f = female)		Age (years)	Ultimate valgus torque (Nm)	Stiffness (N/mm)	Failure load (N)	Elbow flexion angle (degrees)
Ahmad et al. (2003)		10 (10 m)	43 (26–60)	34.0 ± 6.9	42.81 ± 11.6	N/A	70
McGraw et al. (2013)		10 (3 f & 7 m)	52 ± 6	35.0 ± 14.0	21.0 ± 9.0	N/A	30
Hechtman et al. (1998)		31 (N/A)	N/A	22.7 ± 9.0	N/A	N/A	45 or 30
Regan et al. (1991)		8 (6 f & 2 m)	N/A	N/A	N/A	260.9 ± 71.3(AOL) 158.9 ± 4 0.1(POL)	N/A
Dillman (1991)		11	N/A	32.9 ± 5.4	N/A	642 ± 5.4	N/A
Jackson et al. (2016)		6 (1 f & 5 m)	67 (50–83)	N/A	N/A	293.1 ± 38.7(AOL)	70

The contribution of the anterior and posterior band of the AOL to resist an external valgus torque varies with elbow flexion. Two studies have reported that only the anterior band stabilized the elbow in varus-valgus motion over the full range of flexion, whereas the posterior band was a secondary constraint from 90 degrees (Callaway et al. 1997; Floris et al. 1998). More recent studies found that the anterior band showed a constant strain pattern over the elbow flexion-extension range, whereas the strain in the posterior band increased linearly with elbow flexion (Ciccotti et al. 2009; Jackson et al. 2016). In addition, Jackson et al. (2016) found that both bands showed similar intrinsic properties, which indicates the importance of the insertion point and not the intrinsic differences between the anterior and posterior bands (Jackson et al. 2016). Overall, elbow flexion influences how the AOL is loaded. The anterior band of the AOL is important in stabilizing over the full range of flexion, whereas the posterior band seems to have a more stabilizing effect in a flexed elbow.

In all of the studies mentioned earlier, only the study of Jackson et al. (2016) took material fatigue into account. Most measurement protocols started with a preload and increased the load until failure. However, as mentioned before, most of the UCL injuries are overuse injuries and related to fatigue and pitch count. Therefore, it would be useful to take material fatigue of the UCL into account.

### The association between external valgus torque and UCL injuries during pitching

Most research in the field of baseball pitching biomechanics has focused on quantifying kinematic and kinetic parameters across the movement. The net joint torques between segments are calculated by inverse dynamics. In multiple studies, across various levels of pitching and age of the pitcher, the peak external valgus torque has been reported in the range of 45–120 Nm during the late cocking or acceleration phase in the baseball pitch (Werner et al. 1993; Aguinaldo and Chambers 2009; Gasparutto et al. 2016). It has been shown that the peak external valgus torque is lower in youth baseball players (range of 18–27 Nm) (Sabick et al. 2004; Nissen et al. 2007), probably because of lower ball speed, body weight and height.

Some studies have investigated the effect of the external valgus torque in relation to UCL properties. While it is generally assumed that a high external valgus torque around the elbow joint places the UCL under high stress leading to an increased UCL injury risk, only a few studies provide (indirect) support for this assumption. Hurd et al. (2011) found a weak but significant relationship between the external value torque and UCL thickening (r = 0.45 and P = 0.02). Anz et al. (2010) first measured and then subsequently followed 23 professional pitchers for three seasons. The results showed that those pitchers who got injured within the three-season window threw with a significantly higher external valgus torque compared to the non-injured group prior to the follow-up period (Anz et al. 2010). Although these studies investigated the link between external valgus torque and UCL injury and properties, they do not provide information about the UCL loading during a baseball pitch.

### The apparent mismatch between load in in-vitro studies and pitch dynamics

Assuming that both in-vitro studies and in-vivo studies are inherently valid, it can be concluded that there is a mismatch between the ultimate in-vitro valgus torque (34 Nm) and in-vivo peak valgus torque in adolescents (45–120 Nm). If we combine these data, the peak torque in a pitch exceeds the ultimate valgus torque of the UCL by 10–95 Nm. This means that during almost every pitch, the valgus torque of the UCL is exceeded, which raises the question of why ‘only’ 16 out of 100 elite baseball pitchers sustain a UCL rupture during their career.

There are three not mutually exclusive possibilities that contribute to this paradox, namely: underestimation of the in-vitro ultimate valgus torque; overestimation of the in-vivo peak valgus torque; or underestimation of the influence of other torque-resisting structures.

Possibly, the in-vitro ultimate valgus torque is underestimated due to the fact that these studies are done on adult specimens with likely no background in baseball or overhead sports. As a consequence of pitching, the UCL will adapt and thus will be able to resist more loading. On the other hand, and working against the underestimation argument, UCL in-vitro studies have not investigated material fatigue where it is known from the work by Thornton et al. (2015) on rabbits that the knee medial collateral ligament ruptures earlier by fatigue and creep (Thornton et al. 2015).

Overestimation of the peak external valgus torque in-vivo could be due to the assumptions made in inverse dynamic models used such as anthropometric models, coordinate systems and joint centres (Derrick et al. 2019). For example, most inverse dynamics models define the midpoint between the medial and the lateral humerus epicondyle as the joint rotation centre. Moving from the centre to medial or lateral would change the magnitude of the calculated torque. It is, however, mathematically unlikely that this will lead to torque values that are lower than the in-vitro estimate ultimate torques. These model assumptions could also explain the large differences between peak external valgus torques in different inverse dynamic studies (45–120 Nm). If we assume that the study with the lowest external peak valgus torque of 45 Nm in adult pitchers is the ‘true’ value, there is still 10 Nm difference compared to in-vitro studies.

The third option, underestimation of the effect of other structures in-vivo is most likely the explanation for the difference between in-vivo and in-vitro data. The in-vivo torque is calculated as the resultant joint torque, which also includes the possible contributions of muscles and joint articulations and should thus in fact not be solely attributed to the UCL. To really quantify the UCL injury risk, these factors should be considered.

## Structural and functional elbow stabilizers

### Structural stabilizers

When an elbow resists valgus torque, a compression force on the lateral side, between the radial head and the humerus occurs. In mechanical terms, a compression force provides stability. Thus, the geometry of the radiohumeral articulation could be related to resist the valgus torque over the full range of motion. Hotchkiss and Weiland (1987) placed thirty elbow cadavers under a valgus torque of 1.3 Nm over 2 seconds. They found that the torque–displacement curve increased by an average of 30% at 0^◦^, 45^◦^ and 90^◦^ elbow flexion, after excision of the radial head. It is important to note that in their study, cutting the UCL resulted in such destabilization of the joint that the torque–displacement curve could not be measured. Morrey et al. (1991) performed comparable tests, with only gravity as applied torque, and found that when the UCL was intact sectioning the radial head did not result in any change in laxity at all. When the UCL was cut, it did result in up to 12.5^◦^ more laxity, pointing to the radiohumeral joint as a secondary stabilizer. An important difference compared to the study of Hotchkiss and Weiland (1987) is that their experimental setup contained three upper arm muscles (biceps, brachialis and triceps), which could increase the compression force and thus stability when the UCL was cut. Another difference between the two studies is that Morrey et al. (1991) only applied a gravitational torque, it might be possible if a dynamic torque was applied, also a laxity was found with an intact UCL. It should be noted that in both studies the applied torque is very low compared to the inverse dynamic valgus torques.

In conclusion, the UCL is important in stabilizing, but next to the UCL also the radiohumeral joint is a structural stabilizer that can resist elbow valgus torque. It seems that the magnitude of contribution depends on the amount of compression force and the magnitude of the externally applied torque.

### Functional stabilizers

Muscles have the potential to function as functional stabilizers in counteracting an external valgus torque. Davidson et al. (1995) started investigating the anatomy of the Flexor Pronator Mass (FPM) muscles, which consist of the flexor carpi ulnaris (FCU), flexor digitorum superficialis (FDS), flexor carpi radialis (FCR) and pronator teres (PT), to identify which muscles lay directly over the UCL in 30°, 90° and 120°of elbow flexion. They found that the FDS and the FCU partially or fully lay over the UCL, whereas the FCR and PT never lay over the UCL. Their conclusion was that the FCU is optimally positioned to provide support to the UCL, although the FDS has a greater size and force potential for valgus stabilization (Davidson et al. 1995). Multiple studies have tried to quantify the contributions of these various muscles to elbow stability in cadavers using different methods with loading and unloading muscles and with intact and released UCL and at different elbow flexion angles (Table 3). The release of FPM muscles tension with a released UCL showed an increased valgus angle only with the forearm in supination (Seiber et al. 2009). Several studies investigated the effect of the individual FPM muscles on the neutral forearm position (Park and Ahmad 2004; Lin et al. 2007; Udall et al. 2009). Park and Ahmad (2004) simulated the muscle loads with nylon cords at 15 N by a released UCL, and it was shown that the FCU had the most substantial contribution, followed by the FDS and FCR, and the PT has the smallest contribution. Lin et al. (2007) share this conclusion: instead of cutting the UCL, they measured the strain of the UCL when loading the different FPM muscles. They found a decreased strain on the UCL. In contrast to these two studies, Udall et al. (2009) adjusted the loading on the individual muscles to its cross-sectional area and concluded the FDS to be the most significant contributor to valgus stability, followed by a similar contribution of the FCU and the PT (Table 3).

**Table 3.** In-vitro studies that investigated the effect of muscles on resisting external valgus torque. * indicates that the muscle has the potential to resist external valgus torque

	Investigated muscles	Forearm position	Elbow flexion angles	Method	Outcome variable
Seiber et al. (2009)	FPM*	Pronation Supination* Neutral	30 50 70	Elbow loaded with 2 Nm valgus torque and simulated biceps, brachialis and triceps. The passive FPM loading was then released by cutting the tendons.	Valgus angle
Lin et al. (2007)	FCU* FDS* FCR* PT	Neutral	45 90	Muscles were loaded with a free weight pulled a wire that was sutured onto the respective muscles and was loaded individually in degrees by 10 N.	Strain relieve in the UCL (%/10 N)
Park and Ahmad (2004)	FCU* FDS* FCR* PT*	Neutral	30 90	The FPM muscles were individually loaded with a released UCL, and all loaded equally with 15 N. The triceps, biceps and brachialis were loaded by simulated free weights pulling cords.	Valgus angle
Udall et al. (2009)	FDS* FCU* PT*	Neutral	30 60 90	The FDS, FCU and PT muscles were adjusted to its cross-sectional area by 14.4 N, 7.6 N, 8.0 N, respectively, total 30 N. One of the three muscles was unloaded, and three different valgus torques with a max of 1.5 Nm+weight of the forearm was applied.	Valgus angle

**Figure 2.**
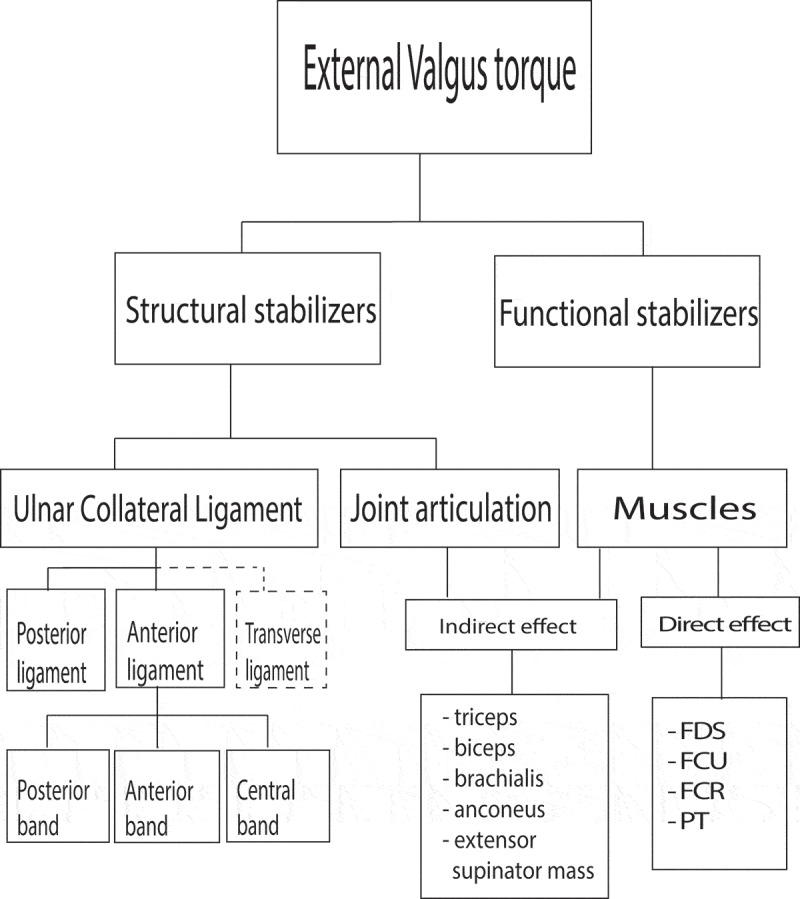
Schematic overview of structural and functional stabilizers which can resist or counteract an external valgus torque according to in-vitro studies. Dashed line: Cannot resist valgus torque, but is part of the Ulnar Collateral Ligament

Fewer studies have discussed the contribution of upper arm muscles to valgus stability. Morrey et al. (1991) showed that simulated functional muscle contributions from the biceps, brachialis and triceps reduce the valgus-angle. Similarly, Seiber et al. (2009) simulated these muscle contributions with nylon lines attached to the tendons near the insertion of these muscles. A load of 20 N was applied to the triceps nylon line and 10 N each to the biceps and brachialis nylon lines. The release of these muscles resulted in an increased valgus-angle. This result could be explained by the effect of the compression force on valgus stability. Due to the co-contraction of the flexor and extensor muscles, a compression force in the elbow is present, but these muscles cannot provide compression force when they are inactive. Next to the upper arm muscles, also the forearm muscles might have an indirect effect due to co-contraction like the extensor supinator mass in relation to the FPM. Hence, the triceps, biceps, brachialis, anconeus and extensor pronator mass muscles cannot provide direct valgus stability, but could possibly have an indirect effect by providing a compression force in interaction with the joint articulation (Figure 2).

### Shielding effects of elbow stabilizers during pitching

Although validation of musculoskeletal models is difficult, these models can provide insight into the combined role of the functional and structural stabilizers during pitching. Experimental EMG studies can partly validate these musculoskeletal modelling studies. Therefore, both experimental and musculoskeletal modelling studies should be performed to investigate the shielding effects of elbow stabilizers.

**Figure 3.**
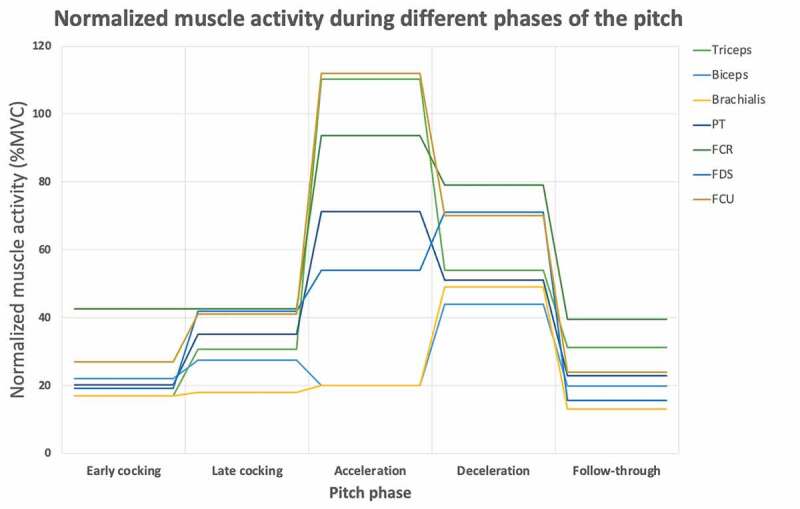
Muscle activity over different phases of the pitch cycle. The lines represent the muscle activity normalized by the maximal voluntary contraction (MVC). The muscle activity is the mean over all (two or three) studies which measured the specific muscle (Sisto et al. 1987, Jobe et al. 1984 & Digiovine et al. 1992)

Electromyography (EMG) studies have the potential to study the effect of muscle stress shielding for the UCL. Sisto et al. (1987) recorded the EMG of eight forearm muscles. They found that FDS, FCR and PT had low-to-moderate activity throughout the pitch. The peak activities occurred in the late cocking phase (30%, 28% and 25% of their maximal voluntary contraction (MVC), respectively). In contrast, Digiovine et al. (1992) found that the peak activity of FDS, FCR, FCU and PT all occurred in the acceleration phase (80%, 120%, 112% and 85% of their MVC, respectively). In high-intensity motions, values over 100% isometric MVC are not uncommon (Ball and Scurr 2013). These values likely indicate that pitchers can recruit more motor units during an explosive pitching movement than during a static MVC test. In the late cocking phase, the phase of maximum valgus loading, their activity levels were also high (40–50% MVC). Jobe et al. (1984) found that the triceps were highly active during these phases and the biceps minimally. Most of the elbow muscles are biarticular, which means that movement around another joint influences the muscle activation. This has no influence on the stabilizing effect because the muscle activity will, due to its joint compression force, have a stabilizing effect around the elbow, irrespective of the movement it is aiming to induce. Figure 3 shows the normalized muscle activity during the different pitch phases. The muscle activity is the mean over all (two or three) studies which measured the respective muscle.

Werner et al. (1993) combined the valgus torque with EMG measurements during pitching. They did not normalize muscle activity, which makes it hard to determine the relative contribution of each muscle. Based on the patterns, they found that the FPM, as well as the anconeus and triceps, was active during peak valgus torque and concluded that the FPM could provide varus torque, while the anconeus and triceps may have helped in minimizing UCL load by compressing the joint. This is in line with the in-vitro studies of Seiber et al. (2009) and Morrey et al. (1991).

If we assume a shielding effect of the functional stabilizers, the timing of the functional stabilizers is crucial. Unfortunately, all EMG studies provided results that were summarized over the throwing phases and are thus not accurate enough to draw conclusions at which instant the muscles studied actually contribute to reduce UCL stress (Figure 3). Preferably, future EMG research should investigate muscle onset timing in more detail, linking kinematics and kinetics time series.

With the upcoming trend of musculoskeletal modelling, it has become feasible to estimate UCL loading, given a sufficiently accurate elbow model. However, up to now, only one published study (Buffi et al. 2015) has investigated the baseball pitch with musculoskeletal modelling. They used an open-source musculoskeletal model with 14 elbow internal varus muscle-tendon actuators to forward dynamic simulate the baseball pitch. The maximum external valgus torque imposed on the upper arm throughout the pitching motion was 115 Nm. From the simulations, it appeared that the FDS could have the most extensive contribution to counteract the valgus torque, followed by the PT and the FCR, although the model showed that activity appears 40 ms after peak valgus torque, probably around the instant of ball release, which is later compared to the rough EMG results. The triceps had the largest contribution during external peak valgus torque. It worked out to be impossible to create enough muscle force to counteract the external torque and the osseous and/or UCL contributions were also needed. A drawback of the model was the difficulty of combining the ligamentous and muscular contribution in the model, which is a generally recognized limitation of musculoskeletal models to date.

## Conclusion

The goal of this review was to provide an overview of what risk factors are related to UCL injuries and to better understand the relationship between the UCL properties and elbow stabilizers with the load on the UCL during pitching, by combining the literature of in-vitro and in-vivo studies. In-vitro studies show that the ultimate UCL torque is around 35 Nm, whereas in-vivo studies found higher peak valgus torques of 120 Nm during pitching. This mismatch raises the question of why ‘only’ 16% of the pitchers sustain a UCL injury. The explanation for this mismatch is most likely the underestimation of elbow structures, among which structural and functional stabilizers in inverse dynamic models. In-vitro studies demonstrate the direct UCL shielding potential of the FPM muscles and indirect interaction of elbow flexor-extensor muscles with the compression force of the joint geometry. EMG studies show muscle activity of the FPM and elbow flexor-extensor muscles during pitching. However, these results are summarized over pitch phases and are therefore not sufficiently accurate to conclude on a UCL shielding effect. Musculoskeletal models show potential to investigate the effect of joint geometry, next to the muscles. However, the validation of these models is difficult. Future studies should investigate how the external valgus torque is distributed over the UCL and other stabilizers, to quantify the UCL load during pitching.
